# Rehabilitation of a patient with extensive odontogenic myxoma treated conservatively: A case report with 14 years of follow-up

**DOI:** 10.4317/jced.62679

**Published:** 2025-04-01

**Authors:** Ana L L M Paz, Cristhiane A Leite, Bruno A L A Mariz, Luiz E R Volpato, André C Rocha

**Affiliations:** 1 School of Dentistry, University of Cuiabá, Cuiabá, MT, Brazil; 2 Department of Dentistry, Mato Grosso Cancer Hospital, Cuiabá, MT, Brazil; 3 Hospital Vila Nova Star, São Paulo, SP, Brazil; 4 Hospital Sírio-Libanês, São Paulo, SP, Brazil; 5 Clinical Hospital of the University of São Pulo, São Paulo, SP, Brazil; 6 Hospital AC Camargo Cancer Center, São Paulo, SP, Brazil

## Abstract

Odontogenic myxoma is an odontogenic tumor of mesenchymal origin that presents locally invasive behavior. Its treatment is surgical, and the approach can be more conservative or radical. This article presents the rehabilitation of a patient with extensive odontogenic myxoma in the mandible treated conservatively with follow-up for 14 years. A 17-year-old male patient sought care with a painless swelling in the mandibular region causing facial asymmetry. Imaging exams showed a mixed image, with intralesional septa, involving the left anterior and posterior regions of the mandible with a small remaining basilar and lingual bone. An incisional biopsy was performed which confirmed the diagnosis of odontogenic myxoma. Due to the extent of the lesion, a conservative approach was chosen, with excision of the lesion and peripheral ostectomy, with recurrence of the lesion after 48 months, with a new approach being performed. After 14 years of follow-up, without further recurrence of the lesion, the patient was rehabilitated with dental implants and protocol-type prosthesis. Conservative treatment led to bone remodeling and enabled patient rehabilitation with oral implants and an implant-supported prosthesis. Thus, conservative treatment of odontogenic myxomas is a viable alternative for similar cases, and its longitudinal postoperative follow-up is essential.

** Key words:**Conservative Treatment, Dental Implants, Myxoma; Oral Pathology, Oral Surgery.

## Introduction

Odontogenic myxoma (OM) is a rare, locally aggressive, odontogenic tumor of benign mesenchymal origin. It mainly affects adults in the second and third decades of life but can occur at any age; it has a slight predilection for females and can occur in the maxilla and mandible, the latter being affected in two thirds of cases (1,2). Mandibular lesions are most found in the molar and premolar areas and often extend to the ramus. Clinically, most OM are painless, slow growing, and are discovered during a radiographic examination (3).

There is controversy over the most appropriate treatment modality for OM. A more conservative approach involves enucleation and curettage of the lesion, aiming to preserve function and maintain uninvolved structures, reserving radical resection for cases of recurrence (4). OM can present aggressive behavior and high recurrence rates with an average of 25%, thus the understanding of how the treatment modality influences the prognosis is still being discussed (2).

This article presents the case of a patient with extensive odontogenic myxoma in the mandible treated in an unconventional conservative manner, posteriorly rehabilitated with osseointegrated dental implants and protocol-type prosthesis.

## Case Report

A 17-year-old male patient reported an asymptomatic swelling with a four-year evolution. On extraoral physical examination, facial asymmetry was observed due to expansive growth involving the symphysis and left mandibular body, painless on palpation. On intraoral examination, there was effacement of the gingival-labial sulcus with buccal and lingual bulging and displacement of the mandibular anterior teeth.

**Figure 1:**
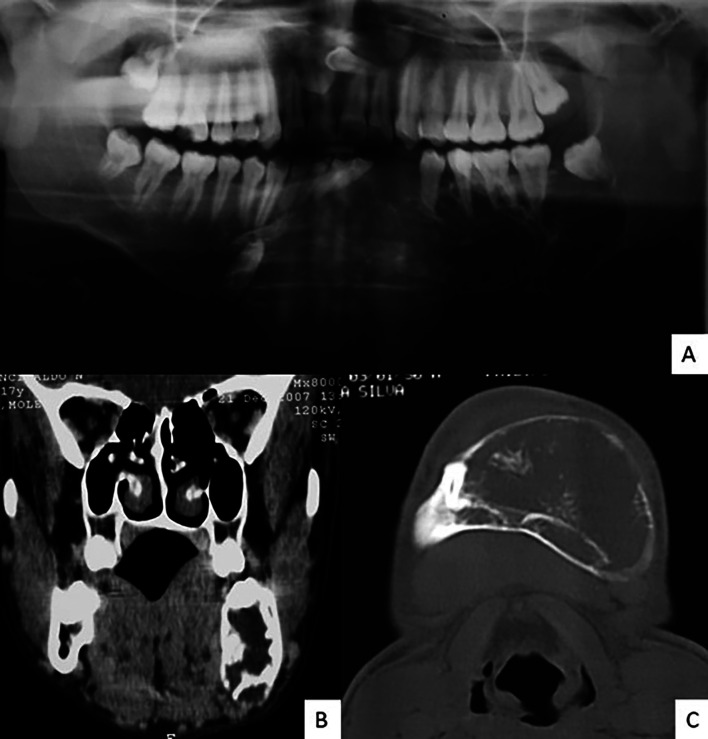
A) Panoramic radiograph showing a multilocular image, poorly defined, extensive in the bilateral anterior and posterior regions of the mandible on the left, radiolucent with radiopaque intralesional trabeculae, displacement of mandibular anterior teeth on the right side, tooth 33 retained in the right mandibular base, root resorption of teeth 34, 36 and 37. Computed tomography in B) coronal section showing hypodense image causing bone expansion in the posterior mandibular region on the left with bone fenestration in the lingual and basal cortical; and C) axial section showing multilocular hypodense image causing mandibular expansion, with hyperdense areas inside, with preservation of the lingual cortex.

In the panoramic radiography, it was possible to observe a radiolucent and multilocular image, extending from the right mandibular body to the left mandibular ramus and from the alveolar bone crest to the base of the mandible, causing deformity and discontinuity. Tooth displacement and inclusion of the mandibular left canine in the tumor mass were also noted. On computed tomography, coronal and axial sections, an extensive mandibular lesion with a hypoattenuating aspect in relation to the musculature and the presence of delicate bone trabeculae was observed, causing fenestration in the basal cortex, bulging and expansion of the buccal cortex and preservation of part of the lingual cortex. The diagnostic hypotheses were odontogenic myxoma and conventional ameloblastoma (Fig. 1).

**Figure 2:**
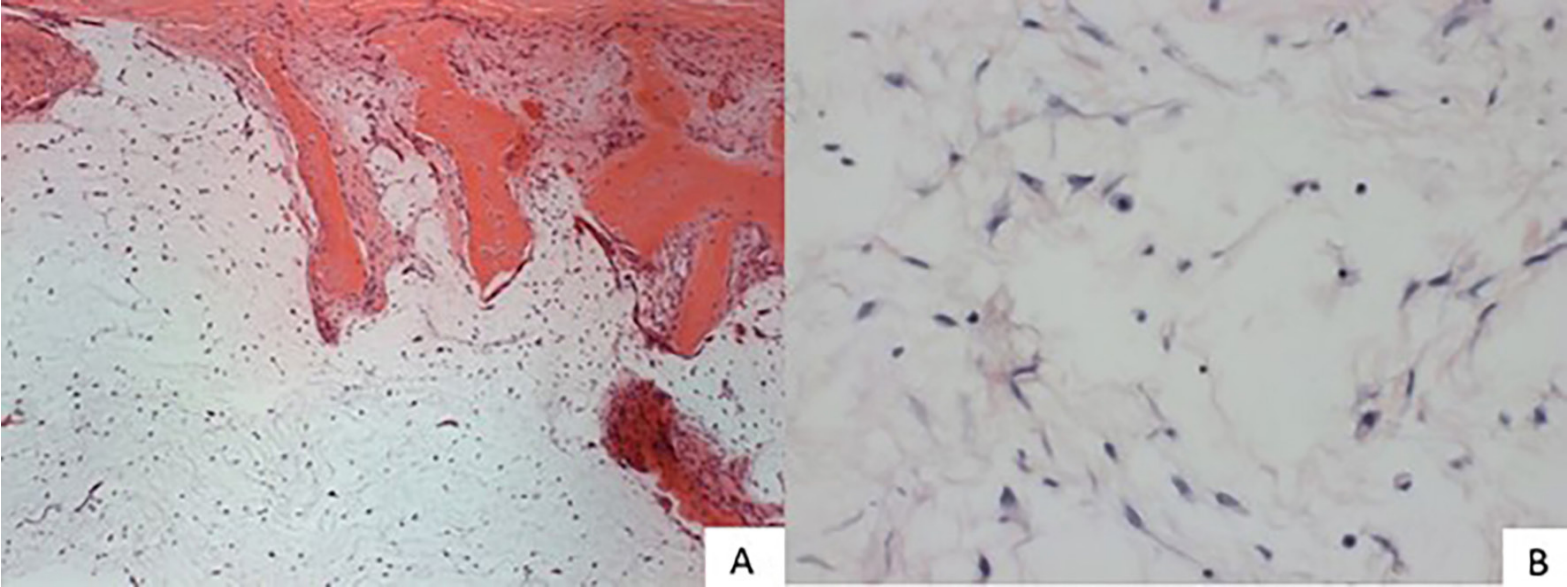
A) A myxomatous proliferation is observed infiltrating the bone trabeculae composed of stellate fusiform cells randomly organized in an abundant loose stroma composed of few thin collagen fibrils (100 times magnification). B) Fusiform and stellate cells can be seen in greater detail, showing oval basophilic nuclei with loose chromatin and pale eosinophilic cytoplasm. The presence of mast cells in the loose stroma is also observed (200x magnification).

An incisional biopsy was performed, which demonstrated myxomatous proliferation infiltrating the bone trabeculae composed of organized stellate fusiform cells establishing the diagnosis of odontogenic myxoma (Fig. 2).

Despite the primary indication for mandibular resection for standard treatment of the case, due to the impossibility of adequate reconstruction for this type of defect and for the preservation, even if exiguous, of parts of the lingual and basal cortex, we opted for the excision of the lesion by intraoral access. The patient was submitted to general anesthesia, nasotracheal intubation, intrasulcular access and relaxant with a number 15 scalpel blade, mucoperiosteal detachment, maintaining the plane of separation between the lesion and the submucosal tissue, vestibular ostectomy to access the entire lesion, excision of the lesion and the teeth involved, curettage and peripheral ostectomy, followed by suturing of the mucosa.

**Figure 3:**
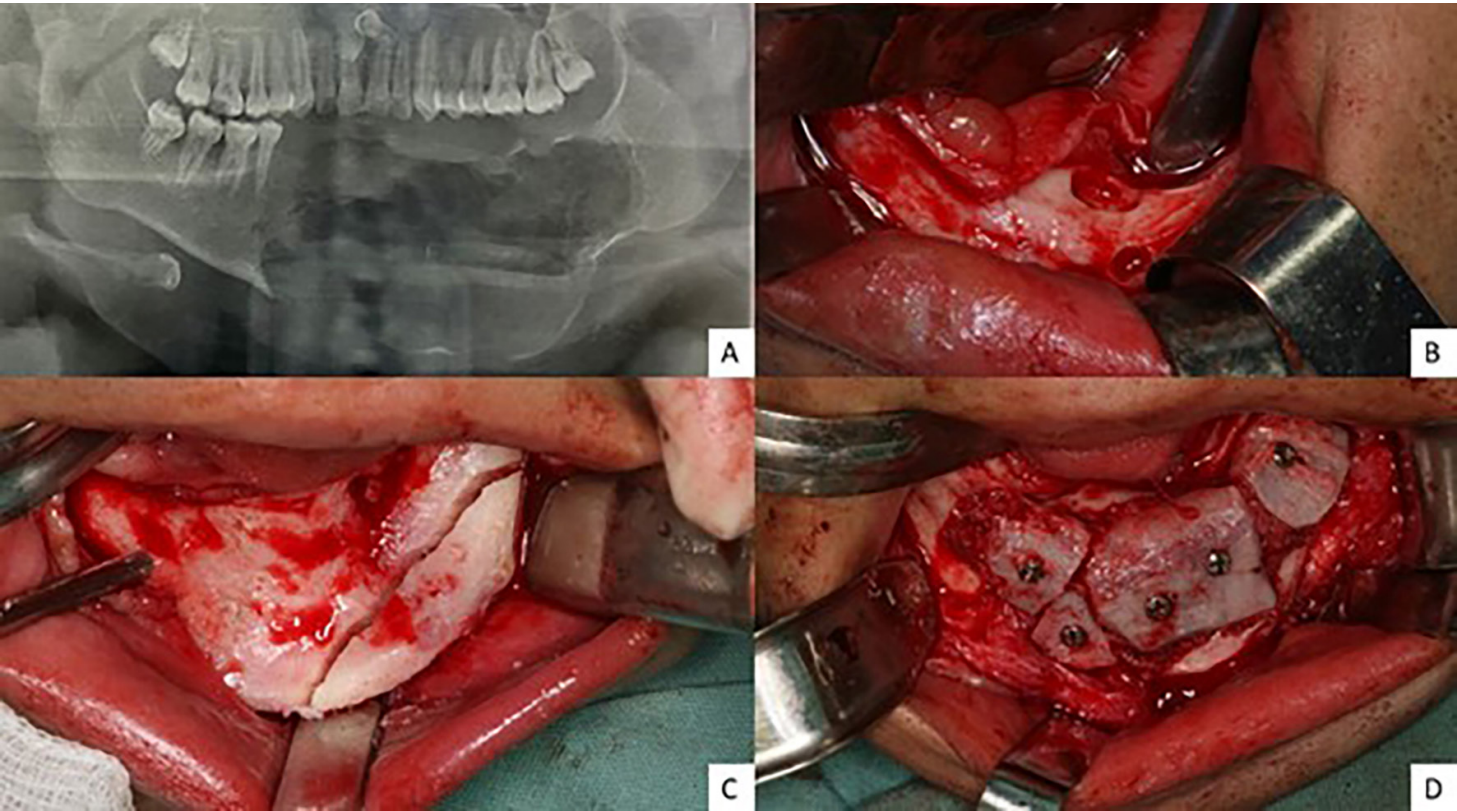
A) Panoramic radiography showing well-circumscribed unilocular images in the left basilar region. Trans-surgical images showing B) pockets in the mandibular region after curettage of a residual lesion; C) mandibular osteoplasty and D) adaptation of an iliac crest bone graft with graft screws in the mandibular region.

After three years of follow-up, bone remodeling was observed with well-circumscribed unilocular images, predominantly in the left basilar region, suggestive of residual lesion. As a result, a new surgical procedure was programmed to remove the lesion, improve the facial contour, and attempt to increase bone thickness with an iliac crest graft (Fig. 3). However, there were signs of contamination and non-incorporation of the graft, which was removed after 40 days, under local anesthesia.

**Figure 4:**
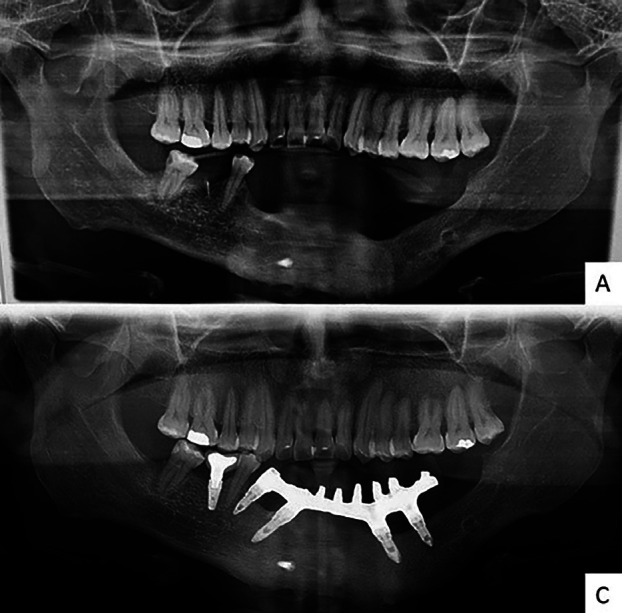
Panoramic radiographs showing A) follow-up of 120 months with bone remodeling without residual lesion. B) Five osseointegrated dental implants in the mandibular region and radiopaque material compatible with metallic bar of protocol prosthesis.

After 120 months of follow-up, showing signs of bone remodeling and no recurrence of the lesion, it was proposed to install osseointegrated implants and a protocol-type prosthesis (Fig. 4). Restoring masticatory, phonetic and aesthetic functions in a more adequate way.

## Discussion

We present the case of a patient with a large odontogenic myxoma in the mandible, with a formal indication for treatment by segmental mandibulectomy and reconstruction with a microsurgical fibula flap. However, due to structural issues and the presence of exiguous basal and lingual cortical bones, conservative treatment was chosen. There was persistence of part of the lesion, which was re-approached after 48 months. The patient was rehabilitated with dental implants and protocol type prosthesis and is currently being followed up with no evidence of disease.

At the time of diagnosis, the patient was 17 years old, within the age group most affected by OM, which is the second and third decades of life (5,6). However, he is a man, less prevalent sex (1,6,7).

OM is a slow-growing benign tumor, thus remaining asymptomatic (1,3) as in the presented case. The increase in volume is a common complaint among patients with OM (1) and was present in the case.

In this case, the OM was in the mandible, the most affected site (2). Radiographically, the lesion presented as a multilocular image with ill-defined margins involving teeth with displacement and resorption. The radiographic features of OM can range from small, radiolucent unilocular images to extensive multilocular lesions with well-defined or diffuse margins and a thin trabecular bone within its structure resembling “honeycomb”, “soap bubbles” or “tennis racquet” (8). In some cases, the involved teeth may show resorption, displacement and a combination of resorption and displacement (9).

The treatment modality for OM is a controversial topic due to its benign nature, but with high recurrence rates, often attributed to incomplete removal of the lesion that has a gelatinous and friable consistency (10). The decision for a conservative approach can be influenced by multiple factors, such as the size and clinical behavior of the lesion, location, patient age and surgeon experience (11). For young patients, conservative treatment can be adopted as a first-line treatment modality for OM, however, follow-up of patients with OM should be prolonged, especially in the first two years (10).

En bloc resection is indicated as the treatment of choice for extensive lesions (12) or for cases that failed to cure after conservative management (13). Conservative management of OM by excision and curettage with liquid nitrogen cryotherapy is an alternative to segmental resection (14). In the case reported, although the patient presented a lesion of extensive proportion, having initially indicated radical treatment with mandibular resection, due to the lack of accessibility to adequate reconstruction for the case, it was decided to perform surgical treatment with intraoral access and excision of the lesion in a conservative way. The lesion persisted after 48 months of follow-up, and a new surgical procedure was performed with local curettage, facial contour improvement and an attempt to increase bone thickness, which failed due to graft contamination, requiring removal. The evolution of the case showed that this reconstruction was not essential, not even to enable implant placement.

The criterion used by the surgeon to make the decision between a radical treatment (block resection) and a conservative treatment (excision, curettage and peripheral ostectomy) involved the presence of the remaining basal and lingual mandibular cortical bone (even though exiguous), which provided a basis for mandibular restructuring, dispensing the use of osteosynthesis material for mandibular reinforcement. In this way, the lesion could be removed using a relatively simple procedure, without the need for extensive resection, which poses a great reconstructive and rehabilitative challenge.

Mandibular defects in patients who underwent resections cause severe bone and soft tissue deformities, with their consequent esthetic and functional sequelae, therefore, immediate mandibular reconstruction is recommended. However, it is a high-cost treatment that is not available for all patients and treatment centers. Mandibular segmental defects not properly reconstructed lead to malocclusion, mandibular deviation, and soft tissue retraction (15). It is known that reconstruction is a challenge for the surgeon, especially when there are limited resources available at the place of work. Thus, conservative treatment, when possible, is a way of helping the patient to treat the disease, maintaining facial contour, occlusal stability and maintaining as many important structures as possible for aesthetics and orofacial function (10).

In the case presented, there was a preference for conservative treatment, even expanding the formal indications of this therapeutic modality, especially in extensive lesions that involve the lower border of the mandible. This conservative approach can help to avoid extensive resections, which, even when accompanied by adequate reconstructive surgeries, are associated with high morbidity procedures. Careful surgical technique and learning curve are essential for the treatment of patients with this approach (2).

It has been suggested that OM recurrence rates are associated with multilocular imaging features, absence of a capsule around the lesion, and large OM treated conservatively (9). The OM in the case presented had a large proportion, was treated conservatively, and presented recurrence. The recurrence of the lesion, however, was not considered a failure of the treatment, since despite the necessary second intervention, it was possible to maintain the initial treatment proposal. Even so, the case presented reinforces the need for longitudinal follow-up of OM cases, especially those treated conservatively, given their high recurrence rate.

## Conclusions

The reported case encourages conservative treatment of odontogenic myxomas of the mandible, extending this indication even to larger lesions, as long as some cortical reinforcement remains. Longitudinal postoperative follow-up is essential in cases of conservative treatment of odontogenic myxomas.

## Data Availability

The datasets used and/or analyzed during the current study are available from the corresponding author.
